# Comments on: Clarification regarding data extraction in the meta-analysis of prenatal acetaminophen exposure and autism

**DOI:** 10.61622/rbgo/2026rbgo108L2

**Published:** 2026-09-23

**Authors:** Pedro do Valle Teichmann, José Paulo de Siqueira Guida, Maria Celeste Osório Wender

**Affiliations:** 1 Pontifícia Universidade Católica do Rio Grande do Sul Porto Alegre RS Brazil Pontifícia Universidade Católica do Rio Grande do Sul, Porto Alegre, RS, Brazil.; 2 Universidade Federal de Ciências da Saúde de Porto Alegre Porto Alegre RS Brazil Universidade Federal de Ciências da Saúde de Porto Alegre, Porto Alegre, RS, Brazil.; 3 Universidade Estadual de Campinas Departamento de Tocoginecología Campinas SP Brazil Departamento de Tocoginecología, Universidade Estadual de Campinas, Campinas, SP, Brazil.; 4 Universidade Federal do Rio Grande do Sul Departamento de Ginecologia e Obstetrícia Porto Alegre RS Brazil Departamento de Ginecologia e Obstetrícia, Universidade Federal do Rio Grande do Sul, Porto Alegre, RS, Brazil.

Dear Editor,

We read with great interest the systematic review and meta-analysis by Oliveira et al. evaluating prenatal acetaminophen exposure and autism spectrum disorder (ASD).^(1)^ We congratulate the authors for addressing an important and controversial topic.^(2)^ However, we would appreciate clarification regarding the effect estimate extracted from the study by Ahlqvist et al.^(3)^ In the pooled analysis of ASD, Ahlqvist et al. contributes approximately 95.6% of the statistical weight, making the overall result highly dependent on the estimate assigned to this single study. The meta-analysis reports an odds ratio (OR) of 1.18 (95% CI 1.15–1.21).^(1)^

However, after reviewing both the original publication and its supplementary appendix, we were unable to identify this estimate among the published analyses. Instead, the primary fully adjusted analysis reported a hazard ratio (HR) of 1.05 (95% CI 1.02–1.08), whereas the sibling-comparison analysis specifically designed to reduce shared familial confounding reported an HR of 0.98 (95% CI 0.93–1.04), suggesting no association between prenatal acetaminophen exposure and ASD.^(3)^

The reported OR of 1.18 appears to be derived from the descriptive frequencies presented in table 2 of the supplementary appendix, rather than from any effect estimate reported by Ahlqvist et al.^(3)^ Because table 2 is a descriptive baseline table and not an inferential analysis, we would appreciate clarification as to whether this value was reconstructed from these data or extracted from a published analysis. If the estimate was reconstructed, this methodological choice could influence the pooled result, particularly given the dominant statistical weight of this study. Moreover, the PRISMA 2020 statement recommends that systematic reviews explicitly describe any methods used to prepare or transform study data before statistical synthesis, including conversions or derivation of effect estimate.^(3)^

We would therefore appreciate clarification regarding: (1) whether the effect estimate from Ahlqvist et al.^ (3)^ was extracted directly from the published analyses or reconstructed from descriptive data; (2) if reconstructed, which methodology was applied; and (3) whether a sensitivity analysis using the published adjusted HRs would materially change the pooled estimate or its interpretation.

A secondary point concerns the risk-of-bias assessment. The review classified the risk of bias due to deviations from intended interventions as low. However, according to the original publication, information on acetaminophen exposure later in pregnancy was available for only 17 of the 25 participating birth cohorts. We would appreciate clarification on how this limitation was considered during the ROBINS-I assessment.^(4,5)^

We congratulate the authors on their contribution and believe that clarification of these methodological aspects would strengthen the transparency, reproducibility, and clinical interpretation of this important review.

## Data Availability

The research data are described in the letter presented.
